# Predicting calvarial morphology in sagittal craniosynostosis

**DOI:** 10.1038/s41598-019-55224-5

**Published:** 2020-01-08

**Authors:** Oyvind Malde, Connor Cross, Chien L. Lim, Arsalan Marghoub, Michael L. Cunningham, Richard A. Hopper, Mehran Moazen

**Affiliations:** 1 UCL Mechanical Engineering, University College London, London, WC1E 7JE UK; 2 Seattle Children’s Hospital, Craniofacial Center, Seattle, WA 98105 USA

**Keywords:** Biomedical engineering, Development

## Abstract

Early fusion of the sagittal suture is a clinical condition called, sagittal craniosynostosis. Calvarial reconstruction is the most common treatment option for this condition with a range of techniques being developed by different groups. Computer simulations have a huge potential to predict the calvarial growth and optimise the management of this condition. However, these models need to be validated. The aim of this study was to develop a validated patient-specific finite element model of a sagittal craniosynostosis. Here, the finite element method was used to predict the calvarial morphology of a patient based on its preoperative morphology and the planned surgical techniques. A series of sensitivity tests and hypothetical models were carried out and developed to understand the effect of various input parameters on the result. Sensitivity tests highlighted that the models are sensitive to the choice of input parameter. The hypothetical models highlighted the potential of the approach in testing different reconstruction techniques. The patient-specific model highlighted that a comparable pattern of calvarial morphology to the follow up CT data could be obtained. This study forms the foundation for further studies to use the approach described here to optimise the management of sagittal craniosynostosis.

## Introduction

Sagittal craniosynostosis is caused by early fusion of the sagittal suture and is the most common form of craniosynostosis^1–6^. Its occurrence rate is about 3 in 10000 birth with several studies reporting a significant increase (2–3 times) in its occurrence in the last 20 years^7–9^. A number of surgical techniques have been developed for the treatment of this condition^10,11^. Many studies have recently compared the clinical outcomes of these techniques in search for the optimum treatment method for this condition^12–14^.

Finite element (FE) method is a powerful numerical technique used to analyse a wide variety of engineering problems^15^ FE method has the potential to predict the morphological changes during the skull growth^16–20^ and to compare the biomechanics of different reconstruction techniques. This can advance our understanding of the optimum management, not only of sagittal synostosis but all forms of craniosynostosis^21–23^. However, FE models first need to be validated and we need to understand the sensitivity of these models to build confidence in their outcomes.

The aim of this study was to develop a validated patient-specific finite element model of a case of sagittal craniosynostosis. Here, the finite element method was used to predict the calvarial morphology of a patient based on their preoperative morphology and the planned surgical techniques. The predicted calvarial morphology was then compared to the *in vivo* computed tomography data two years following the operation. This retrospective study, to the best of our knowledge, is the first study using FE method to predict the outcome of the calvarial reconstruction.

## Materials and Methods

### Patient and image processing

**Figure 1:**
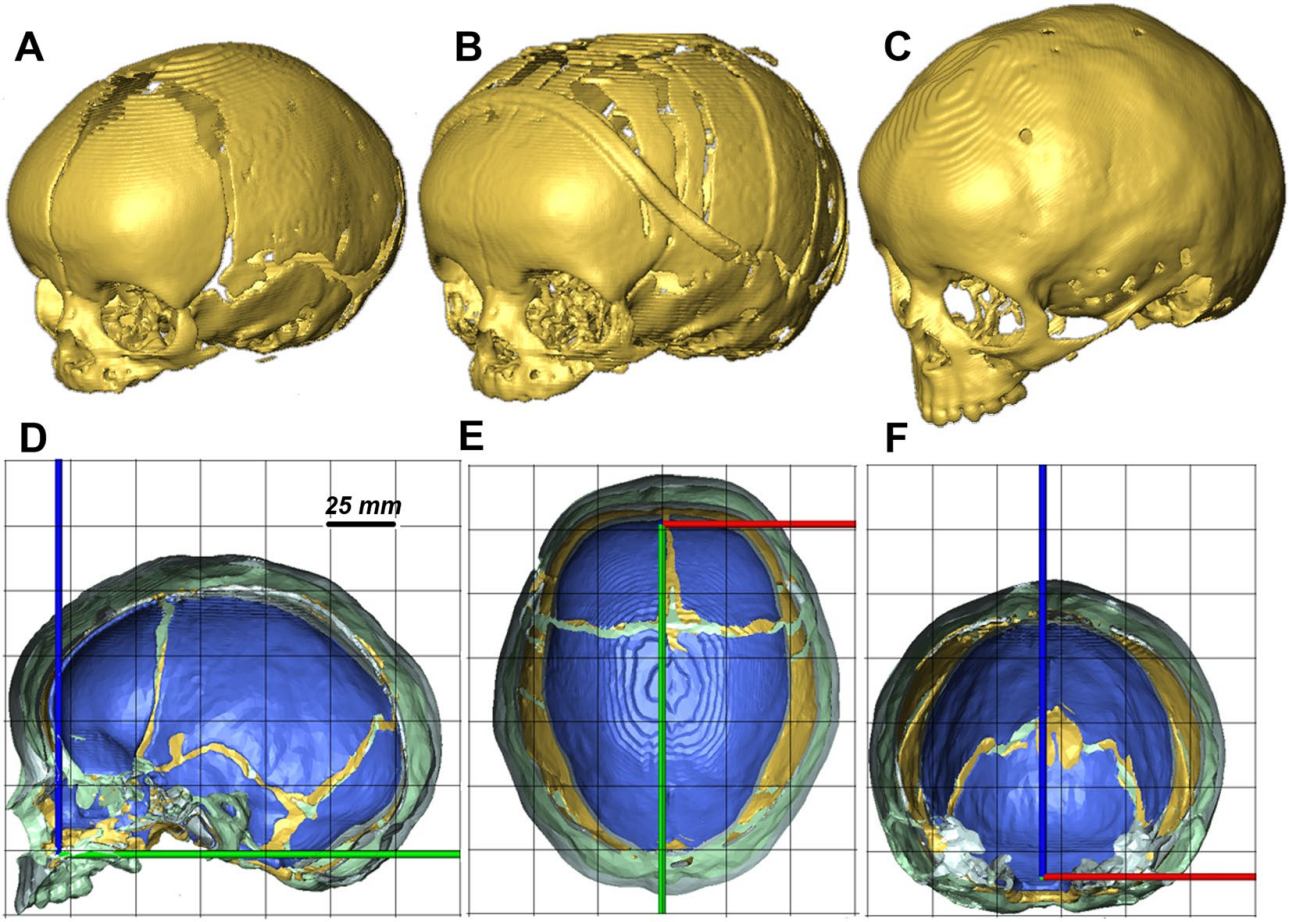
Preoperative (**A**), post-operative (**B**) and follow up (**C**) skull reconstructions of a sagittal synostosis patient. Sagittal (**D**), transverse (**E**) and coronal (**F**) cross-sections of the preoperative (blue), post-operative (yellow) and follow up (grey) skull reconstructions. Note (**A–C**) are not to the scale while (**D–F**) are to the correct scale.

A series of computer tomography (CT) images of a sagittal synostosis patient of unknown sex and identity were obtained from the Seattle Children’s Hospital (Washington, USA). The preoperative CT was obtained at 3 months of age (Fig. 1A); the postoperative CT was obtained at 5 month of age (Fig. 1B) and the follow up CT was obtained at 29 months of age (24 months’ post-operation - Fig. 1C). Figure 1D–F compares the morphological changes of this patient’s skull. Note, this study was reviewed and approved by the Institutional Review Board of Seattle Children’s Hospital (approval number 12394). Written informed consent from the parents or guardians of the child was obtained.

The CT images were imported into Avizo image processing software, (Thermo Fisher Scientific, Mass, USA) and 3D models were developed. Bone, sutures and intracranial volume were segmented on the pre-operative models. The pre-operative model was then reconstructed virtually to model the post-operative calvarial reconstruction. This model consisted of bone, sutures, craniotomies and intracranial volume (ICV) that broadly represent the brain.

### Finite element analysis

#### Model development and materials

The 3D reconstructed pre-operative model was transformed into a 3D solid mesh model and imported to a finite element solver (ANSYS v.18, Canonsburg, PA, USA) to predict the follow up calvarial morphology. A quadratic tetrahedral mesh consisting of 1.6 million elements for the skull/sutures and 200,000 for the ICV was chosen following a mesh convergence study. Isotropic (linear and elastic) material properties were assigned to all regions with a thermal coefficient defined only for the ICV. Bone and suture were assumed to have an elastic modulus of 3000 MPa and 30 MPa respectively^24,25^. The elastic modulus of the ICV was assumed to be 100 MPa^17^. The bone and suture materials were assumed to have a Poisson’s ratio of 0.3. The ICV value was 0.48. The craniotomies were modelled with the same properties as the sutures.

#### Boundary and interface conditions

We made the assumption that the bone-suture and bone-craniotomy were perfectly connected (bonded), and modelled the ICV-bone/suture/craniotomy with contact elements using a penalty-based algorithm. A low tangential friction coefficient of 0.1 was used to represent the frictionless environment at the ICV-bone/suture/craniotomy. Following a series of sensitivity tests similar to the study of Bernakiewicz *et al*.^26^, the normal contact stiffness was set at 500 N/mm and penetration tolerance at 0.5. The sensitivity tests results are included in the supplement (see Supplementary Table S1). These data highlighted that changing the contact stiffness within the range of 25–3000 N/mm, and penetration tolerance in the range of 0.1–0.5, resulted in less than 1% change in the outcome measurements (see Supplementary Table S1).

The model was constrained in all degrees of freedom around the foramen magnum, on the palate and airways. This was similar to our previous study on modelling the natural calvarial growth from 0–12 months of age^17^. The model was loaded via thermal expansion of the ICV, as previously described^17,19^. A linear isotropic expansion was applied to the ICV, where the pre-operative ICV (measured at 648 ml) was expanded to the follow up ICV (measured at 1320 ml) in seven intervals. No adaptive remeshing algorithm was used, as the geometry was updated at each interval to the new deformed shape. This approach avoided element distortions that would have otherwise occurred due to the large deformation.

#### Simulations and measurements

Six simulations were carried out. The main focus is on three key scenarios throughout the study, however a full comparison of the all cases is included in the supplement (see Supplementary Fig. S1). The three key scenarios were:

1. Case 1 – open sutures/craniotomies: mechanical properties of all the sutures/craniotomies were not changed during the growth i.e. from 3 to 29 months of age – a hypothetical scenario;
2. Case 2 – closed sutures/craniotomies: mechanical properties of all the sutures and craniotomies were assigned the same as bone at the 3 months of age scan – a hypothetical scenario;
3. Case 3 –*in-vivo* modelled sutures/craniotomies: suture properties were gradually increased by 200 MPa in seven intervals to take into account the effects of gradual bone formation at the sutures during the development. Here the entire suture elements were selected and their elastic modulus was increased at the end of each interval. The metopic suture was fused at 8.5 months of age as previously described^27,28^. This was intended to model the actual *in-vivo* scenario.

Predicted calvarial morphologies from the simulations were compared against the *in vivo* calvarial morphology at the 29 months of age scan in terms of: (i) cephalic index, i.e. maximum skull width divided by the maximum skull length multiplied by 100, (ii) 2D cross-sections and (iii) 3D distance colour maps. The patterns of contact pressure on the intracranial volumes were also compared as an indication of how each of the considered cases affected the brain growth. Note (1) the changes in the calvarial morphology at each interval is not included here but such results are presented for our previous work on predicting calvarial morphology in mouse and normal human skull growth^17,19,20^. (2) all methods were carried out in accordance with relevant guidelines and regulations.

## Results

**Figure 2:**
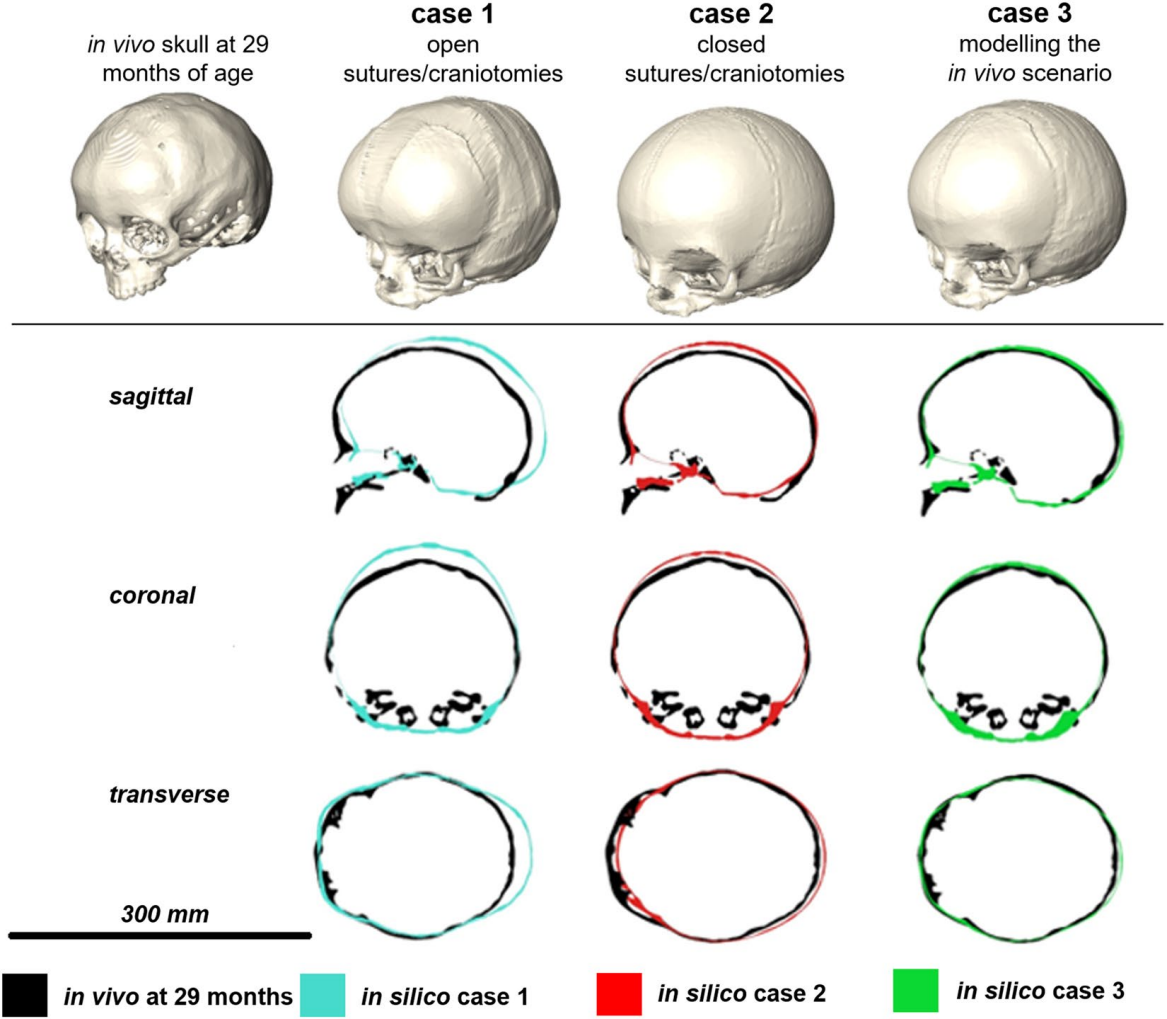
*In silico* cases (1–3) versus *in vivo* follow up skull: sagittal, coronal and transverse cross-sections.

**Figure 3:**
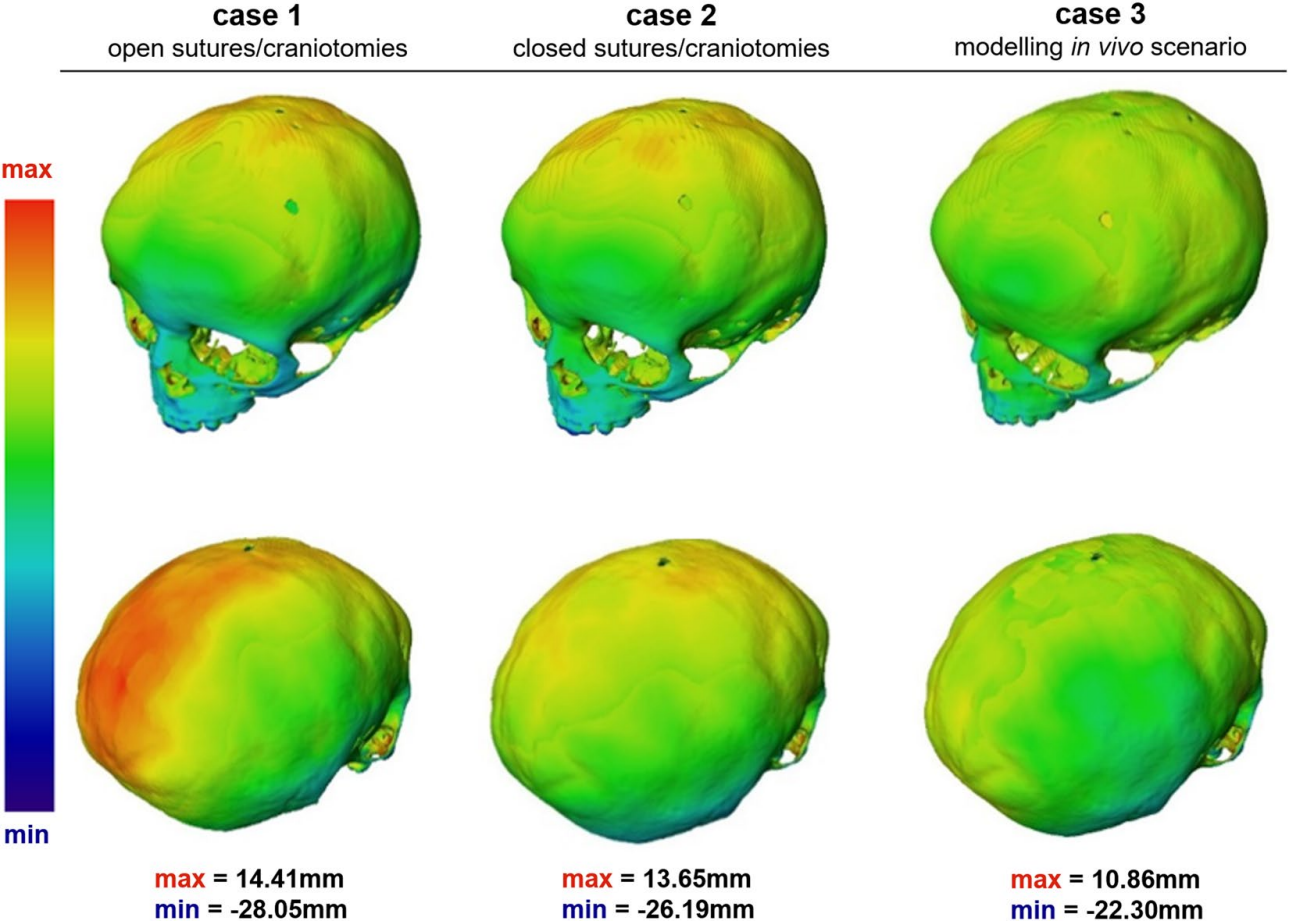
*In silico* cases (1–3) versus *in vivo* follow up skull: three-dimensional distance plots. The red sections highlight where the *in silico* models over-predicted the shape of the *in vivo* skull, while the blue areas indicate where the *in silico* models under-predicted the *in vivo* skull morphology. Each skull has been scaled individually with the maximum and minimum scores for the colour chart given under each case.

The hypothetical Case 1 that assumed open sutures up to 2 years of age, showed the highest difference from the actual *in vivo* scenario. The predicted CI for Case 1 was 0.75 while the *in vivo* CI was 0.83 (Table 1). Similarly, the cross-sectional comparison between this case and the *in vivo* case (Fig. 2) showed that the model over-estimated the posterior growth of the skull. Since the brain growth (i.e. ICV expansion) was not constrained, (i.e. the sutures and craniotomies were patent), the contact pressure at the ICV-bone/sutures/craniotomies was almost negligible across the ICV (i.e. less than 0.1 MPa – see Fig. 3 for Case 1).

**Table 1:** A summary of predicted calvarial measurements and cephalic indexes (CI) of cases 1–3 and the *in vivo* data at 29 month of age or 24 months post-operation.

	Length (mm)	Width (mm)	Height (mm)	CI
*in silico* case 1	173.15	129.84	118.86	75
*in silico* case 2	161.45	132.13	122.04	82
*in silico* case 3	164.23	131.74	114.46	80
*in vivo* at 29 months	167.90	138.87	111.76	83

The hypothetical Case 2 that modelled fusion of all sutures after the operation showed a close match to the CI of the *in vivo* case i.e. 0.82 vs. 0.83 (Table 1). Considering the cross-sectional comparison between the predicated shape of this case and the *in vivo* case, the skull height was over predicted comparing to the *in vivo* result (Fig. 2). Since, all sutures and craniotomies were fused, the contact pressure at the ICV-bone/sutures/craniotomies were much higher than Case 1. There results predicted elevated level of pressure in the anterior part of the ICV, around the orbits.

**Figure 4:**
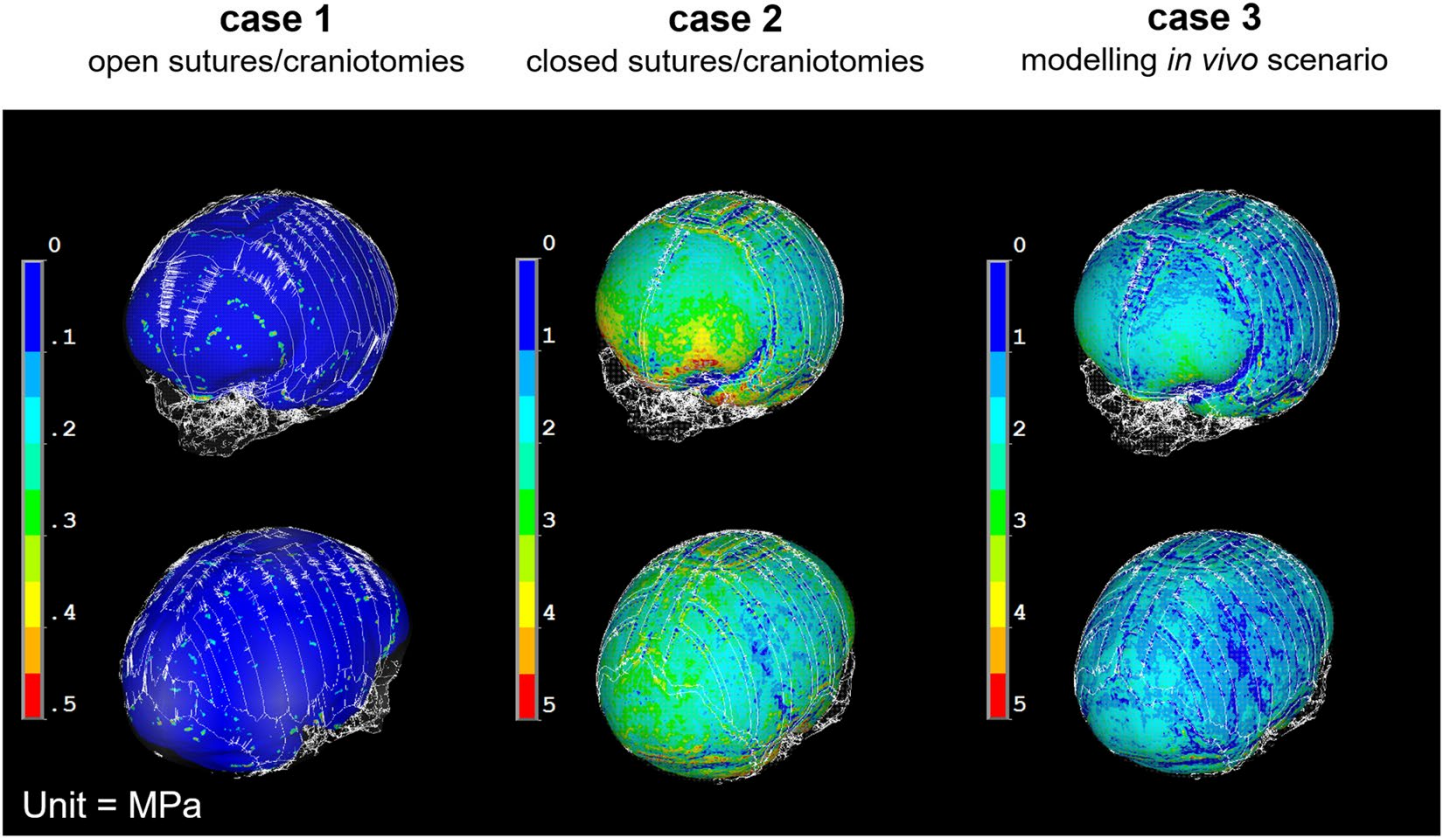
A comparison between the predicted contact pressure on the intracranial volume (brain) between the Case 1–3.

The Case 3 that modelled bone formation at patent sutures most closely matched the actual *in vivo* calvarial growth of the patient two years after surgery. The predicted CI for this case was 0.80 (vs. 0.83 based the *in vivo* data). There was a close match between the predicted skull shape in all cross-sections (Fig. 2) and across the whole skull (Fig. 3). The contact pressure at the ICV-bone/sutures/craniotomies were lower comparing to Case 2 and higher comparing to Case 1 (Fig. 4).

## Discussion

There are limited finite element studies on the biomechanics of craniosynostosis^23^ despite huge potentials of this method to advance treatment of this condition. Our group in the past few years has been using this technique to predict the calvarial growth in humans^17^ and in a mouse model of this condition^19,20^. To the best of our knowledge, the present study is the first attempt to use the finite element method to predict the outcome of calvarial reconstruction in a craniosynostotic patient based on the preoperative CT data.

Three cases were modelled here, two hypothetical (Case 1 & 2) and a more realistic (Case 3) scenario (see Supplementary Fig. S1 for some additional hypothetical cases). The hypothetical cases were aimed to verify the modelling approach and to ensure that it predicts the patterns that are clinically observed or expected. In Case 1, the open suture/craniotomies model, as expected the calvaria expanded with minimal pressure on the ICV; in Case 2, the closed suture/craniotomies model, skull height was increased and there was an elevated level of pressure on the ICV around the orbits, both of which are clinically observed in some of the syndromic forms of craniosynostosis e.g. as clinically observed in Crouzon patients^5,29,30^.

A close match was obtained between the predicted calvarial morphology of Case 3 (modelled *in vivo* bone deposition) and the CT data obtained from the actual patient at 2 years after surgery. Together with the observations in Case 1 and 2, this is reassuring that the modelling approach proposed here has potential to reliably predict the outcome of the calvarial reconstruction. We cannot comment on the validity of the contact pressure maps obtained in this study however, the relative comparison between the three cases are informative.

The modelling approach presented has large potential in predicting calvarial morphology after remodelling surgery and to understand the biomechanical differences between different surgical techniques. In the case of sagittal synostosis this method can be used to compare the existing techniques for the management of this condition and their potential impacts on the brain development. The contact pressure maps that FE models provide us, together with the functional brain imaging data, can advance our understand of the interplay between calvarial reconstruction and brain development^31,32^.

It must be noted that there are other methodologies based on e.g. theories of finite growth and constrained mixtures that have been used to model the growth and remodelling of various living tissues^33,34^. A detail comparison between the approach described here and other theories is beyond the scope of this study. Perhaps one of the key advantages of the approach described here is that it takes into account the interaction between the intracranial volume and the overlying bones, sutures and craniotomies using Hertz contact theory. This is important in the context of calvarial growth and its reconstruction. Nonetheless, the approach presented here has its own limitations.

Perhaps the key limitations of the FE models described here are that: (1) the modelling approach presented here does not directly consider the effects of cerebrospinal fluid and various soft tissues present between the brain and calvarial bones. However, the contact elements used at this interface does take into account to some extent the role of these tissues. Including them explicitly can alter the magnitude of values presented in this study but we believe that the relative comparison between the cases here remains valid; (2) the calvarial reconstruction that was virtually modelled on the pre-operative CT data does not take into account the plastic deformation that may have occurred during surgery. It was evident that there was a difference in the calvarial morphology between the pre and post-operative CT data (Fig. 1). This difference was not taken into account in the models described here. Nonetheless, it is interesting that the model could still predict closely the calvarial shape on two year follow up.

## Conclusions

A validated patient-specific finite element model of calvarial growth was developed in this study. Despite the study limitation, the similarities between the predicted calvarial shape outcomes of the modelling approach and the *in vivo* data are a starting point for future studies. These studies will use the methodology described here to compare biomechanics of different reconstruction techniques and their impact on brain development in sagittal and other forms of craniosynostosis.

## Supplementary information

Supplementary information.
