# Supplementary material for: Predicting calvarial morphology in sagittal craniosynostosis
Source: Sci Rep. 2020 Jan 8;10:3. doi: 10.1038/s41598-019-55224-5 (PMC6949270; doi:10.1038/s41598-019-55224-5)
Supplement: Supplementary file 1 — Supplementary information. [file 41598_2019_55224_MOESM1_ESM.docx]

**Predicting calvarial morphology in sagittal craniosynostosis**

Oyvind Malde^1^; Connor Cross^1^; Chien L Lim^1^; Arsalan Marghoub^1^; Michael L Cunningham^2^; Richard A Hopper^2^; Mehran Moazen^1^

1. UCL Mechanical Engineering, University College London, London, WC1E 7JE, UK; (2) Seattle Children's Hospital, Craniofacial Center, Seattle, WA 98105, USA

**Supplements:**


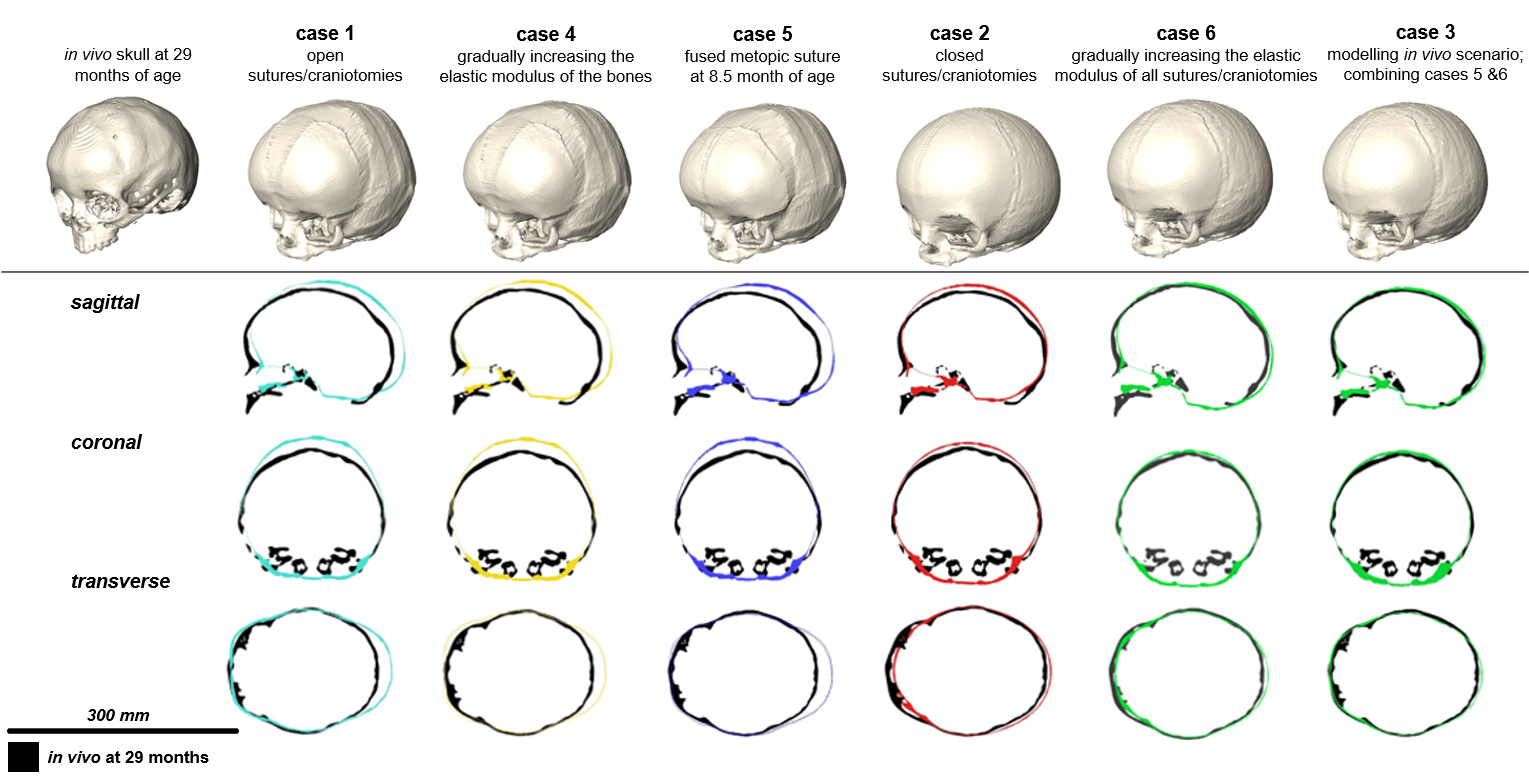


**Fig S1:** *In silico* cases (1-6) versus *in vivo* follow up skull: sagittal, coronal and transverse cross-sections. Case 1-3 are the same that were presented in the main text. Case 4-6 are three additional cases. In Case 4 in addition to the case 1 elastic modulus of the calvarial bones were gradually increased by 250 MPa per step of expansion, equivalent to roughly 2 months. In Case 5, in addition to the case 1, the metopic suture was fused at 8.5 months of age as described by literature.^e.g.27,28^ In Case 6, elastic modulus of the sutures and craniotomies were gradually increased during the calvarial growth in 7 steps with each step representing roughly 2 months of growth. Note Case 3 is a combination of Case 5 and 6.

**Table S1**: Contact parameters sensitivity tests: A summary of the tests i.e. ranges of penetrations stiffness, tolerance, and number of solving steps and compared output for each test.

|  | **Penetration Stiffness (N/mm)** | **Penetration Tolerance** | **Solving steps** | **Length (mm)** | **Width (mm)** | **CI** |
| --- | --- | --- | --- | --- | --- | --- |
| ***in silico*** | 25 | 0.5 | 6 | 173.48 | 129.26 | 74.5 |
|  | 50 | 0.5 | 7 | 172.96 | 129.49 | 74.9 |
|  | 250 | 0.5 | 7 | 173.12 | 129.79 | 75.0 |
|  | 500 | 0.5 | 7 | 173.15 | 129.84 | 75.0 |
|  | 750 | 0.5 | 7 | 173.28 | 130.09 | 75.1 |
|  | 1000 | 0.5 | 7 | 173.09 | 130.12 | 75.2 |
|  | 1500 | 0.5 | 7 | 173.27 | 130.15 | 75.1 |
|  | 2250 | 0.5 | 7 | 172.91 | 129.89 | 75.1 |
|  | 3000 | 0.5 | 7 | 173.01 | 129.92 | 75.1 |
| 500 | 0.1 | 7 | 173.27 | 129.83 | 74.9 |  |
|  |  | 0.2 | 7 | 173.07 | 129.83 | 75.0 |
|  |  | 0.3 | 7 | 173.47 | 130.12 | 75.0 |
|  | 0.4 | 7 | 173.16 | 130.07 | 75.1 |  |
| ***in vivo* at 29 months** | NA | NA | NA | 167.48 | 138.63 | 82.8 |
